# Supplementary material for: Photorhabdus luminescens TccC3 Toxin Targets the Dynamic Population of F-Actin and Impairs Cell Cortex Integrity
Source: Int J Mol Sci. 2022 Jun 24;23(13):7026. doi: 10.3390/ijms23137026 (PMC9266650; doi:10.3390/ijms23137026)
Supplement: Supplementary file 1 [file ijms-23-07026-s001.zip › ijms-1751644-supplementary.pptx]

## Slide 1
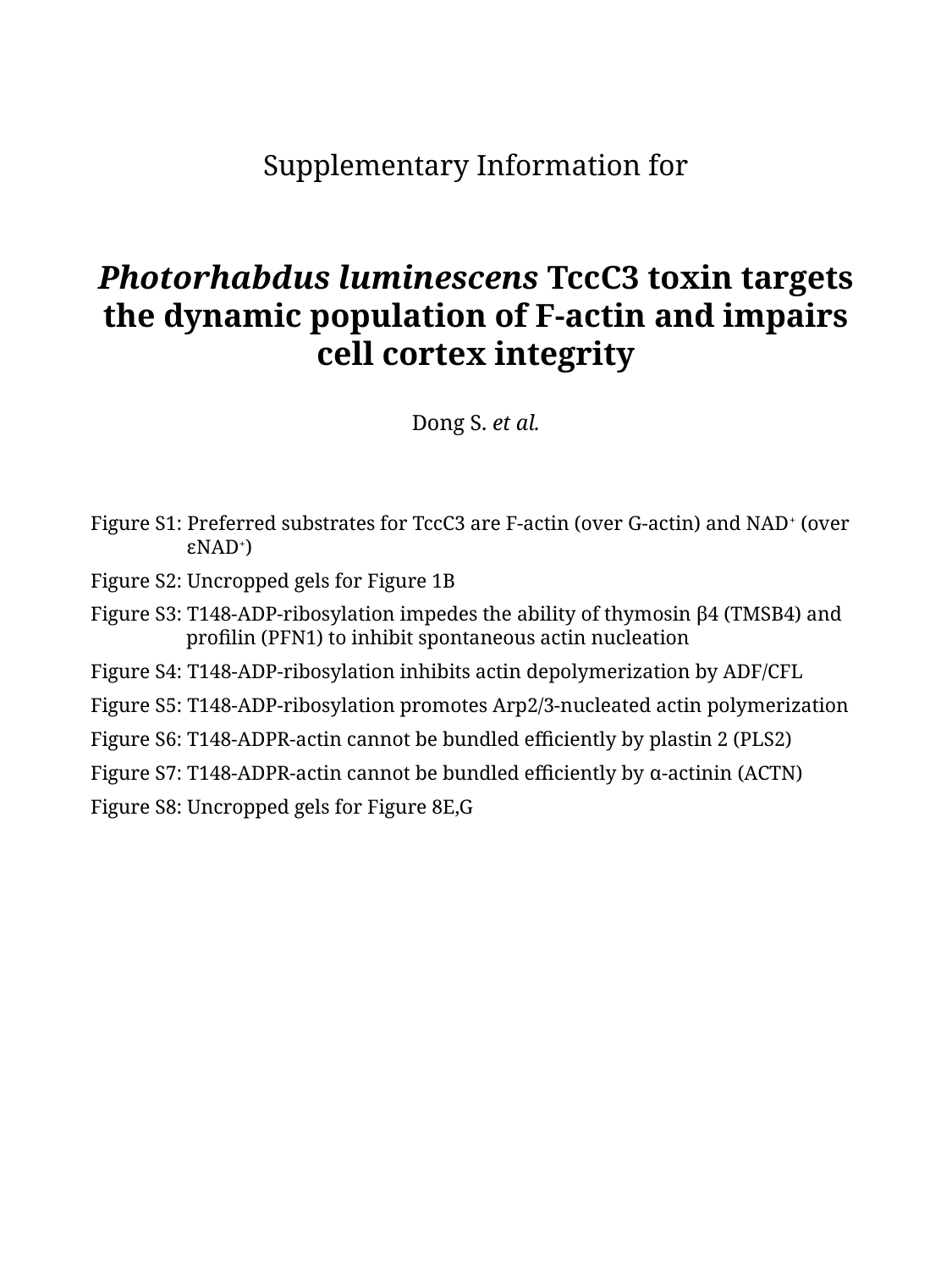

Supplementary Information for
Photorhabdus luminescens TccC3 toxin targets the dynamic population of F-actin and impairs cell cortex integrity
Dong S. et al.
Figure S1: Preferred substrates for TccC3 are F-actin (over G-actin) and NAD+ (over εNAD+)
Figure S2: Uncropped gels for Figure 1B
Figure S3: T148-ADP-ribosylation impedes the ability of thymosin β4 (TMSB4) and profilin (PFN1) to inhibit spontaneous actin nucleation
Figure S4: T148-ADP-ribosylation inhibits actin depolymerization by ADF/CFL
Figure S5: T148-ADP-ribosylation promotes Arp2/3-nucleated actin polymerization
Figure S6: T148-ADPR-actin cannot be bundled efficiently by plastin 2 (PLS2)
Figure S7: T148-ADPR-actin cannot be bundled efficiently by α-actinin (ACTN)
Figure S8: Uncropped gels for Figure 8E,G

## Slide 2
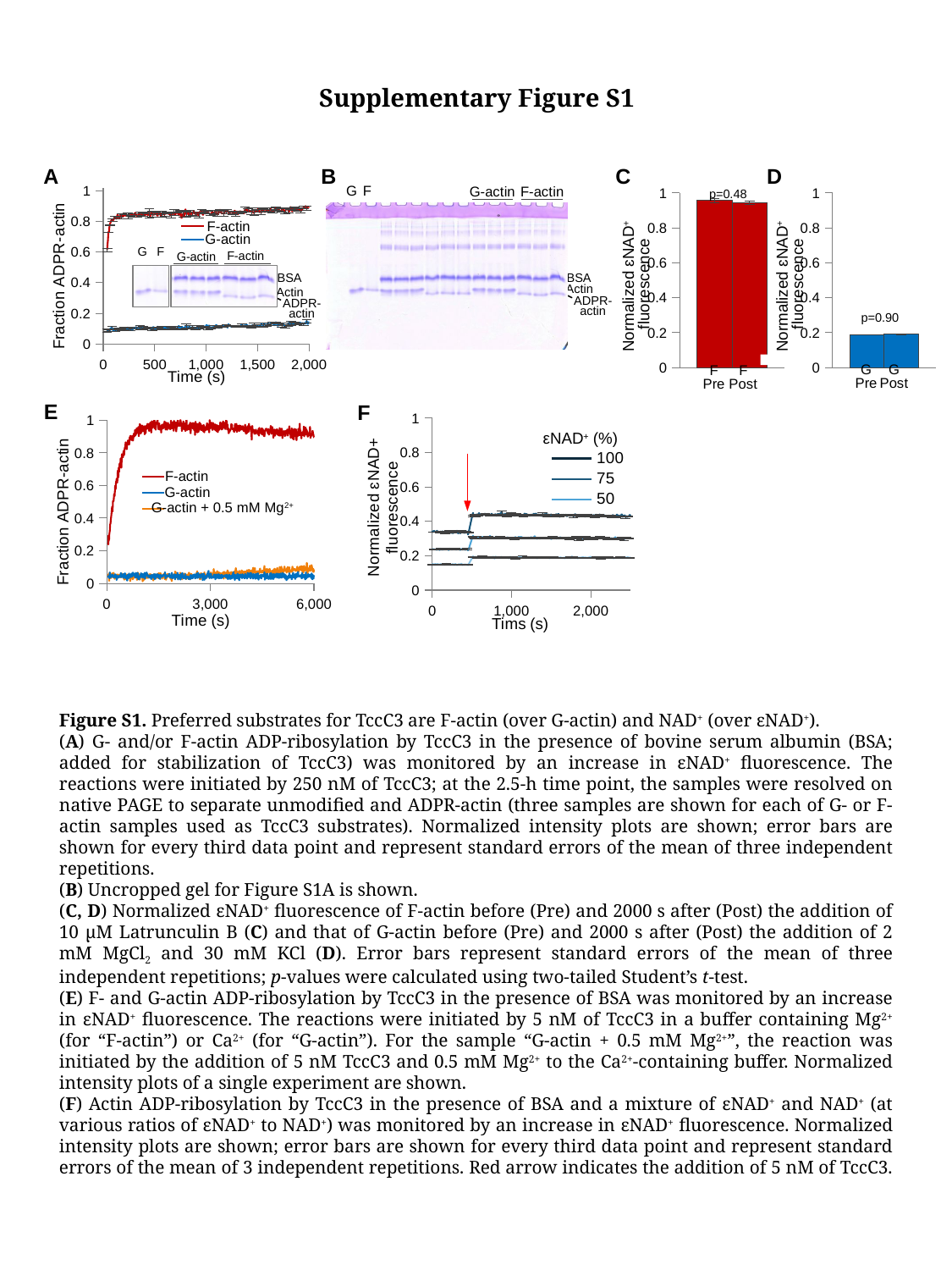

Supplementary Figure S1
A
B
C
D
p=0.48
Normalized ɛNAD+
fluorescence
F
Post
F
Pre
Normalized ɛNAD+
fluorescence
p=0.90
G
Pre
G
Post
G-actin
F-actin
G
F
- BSA
- Actin
ADPR-
actin
### Chart
| Category | F-actin | G-actin |
|---|---|---|F-actin
G-actin
F-actin
G-actin
G
F
- BSA
- Actin
ADPR-
actin
### Chart
| Category | F-Pre | F-Post |
|---|---|---|
### Chart
| Category | G-Pre | G-Post |
|---|---|---|E
F
### Chart
| Category | 100 | 75 | 50 | 25 |
|---|---|---|---|---|εNAD+ (%)
### Chart
| Category | F-actin | G-actin | G-actin+0.5mMMg |
|---|---|---|---|F-actin
G-actin
G-actin + 0.5 mM Mg2+
Figure S1. Preferred substrates for TccC3 are F-actin (over G-actin) and NAD+ (over εNAD+).
(A) G- and/or F-actin ADP-ribosylation by TccC3 in the presence of bovine serum albumin (BSA; added for stabilization of TccC3) was monitored by an increase in εNAD+ fluorescence. The reactions were initiated by 250 nM of TccC3; at the 2.5-h time point, the samples were resolved on native PAGE to separate unmodified and ADPR-actin (three samples are shown for each of G- or F-actin samples used as TccC3 substrates). Normalized intensity plots are shown; error bars are shown for every third data point and represent standard errors of the mean of three independent repetitions.
(B) Uncropped gel for Figure S1A is shown.
(C, D) Normalized εNAD+ fluorescence of F-actin before (Pre) and 2000 s after (Post) the addition of 10 μM Latrunculin B (C) and that of G-actin before (Pre) and 2000 s after (Post) the addition of 2 mM MgCl2 and 30 mM KCl (D). Error bars represent standard errors of the mean of three independent repetitions; p-values were calculated using two-tailed Student’s t-test.
(E) F- and G-actin ADP-ribosylation by TccC3 in the presence of BSA was monitored by an increase in εNAD+ fluorescence. The reactions were initiated by 5 nM of TccC3 in a buffer containing Mg2+ (for “F-actin”) or Ca2+ (for “G-actin”). For the sample “G-actin + 0.5 mM Mg2+”, the reaction was initiated by the addition of 5 nM TccC3 and 0.5 mM Mg2+ to the Ca2+-containing buffer. Normalized intensity plots of a single experiment are shown.
(F) Actin ADP-ribosylation by TccC3 in the presence of BSA and a mixture of εNAD+ and NAD+ (at various ratios of εNAD+ to NAD+) was monitored by an increase in εNAD+ fluorescence. Normalized intensity plots are shown; error bars are shown for every third data point and represent standard errors of the mean of 3 independent repetitions. Red arrow indicates the addition of 5 nM of TccC3.

## Slide 3
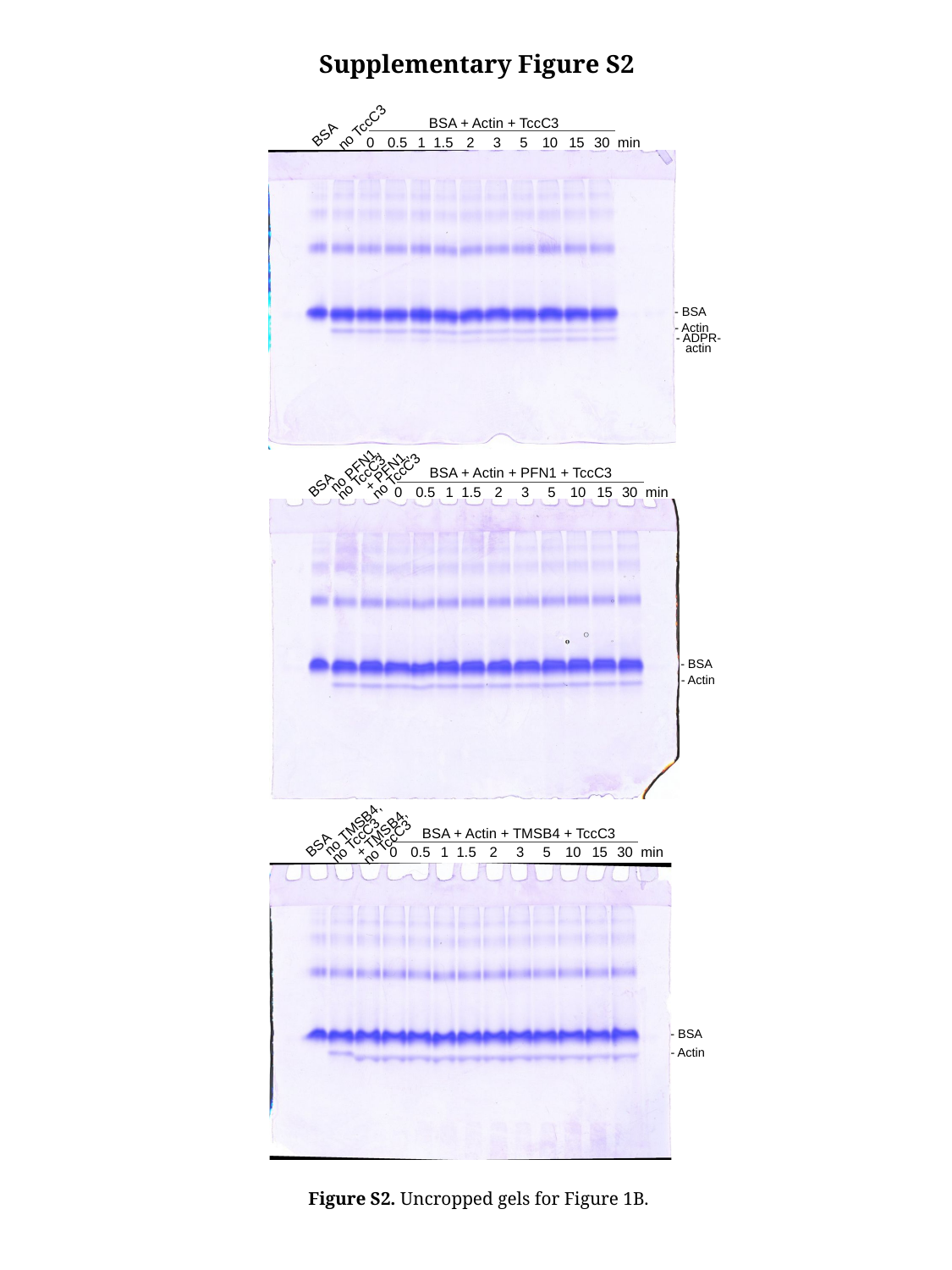

Supplementary Figure S2
BSA + Actin + TccC3
no TccC3
BSA
0
0.5
1
1.5
2
3
5
10
15
30 min
- BSA
- Actin
- ADPR-
actin
+ PFN1,
no TccC3
BSA + Actin + PFN1 + TccC3
no PFN1,
no TccC3
BSA
0
0.5
1
1.5
2
3
5
10
15
30 min
- BSA
- Actin
no TMSB4,
no TccC3
BSA + Actin + TMSB4 + TccC3
BSA
0
0.5
1
1.5
2
3
5
10
15
30 min
- BSA
- Actin
+ TMSB4,
no TccC3
Figure S2. Uncropped gels for Figure 1B.

## Slide 4
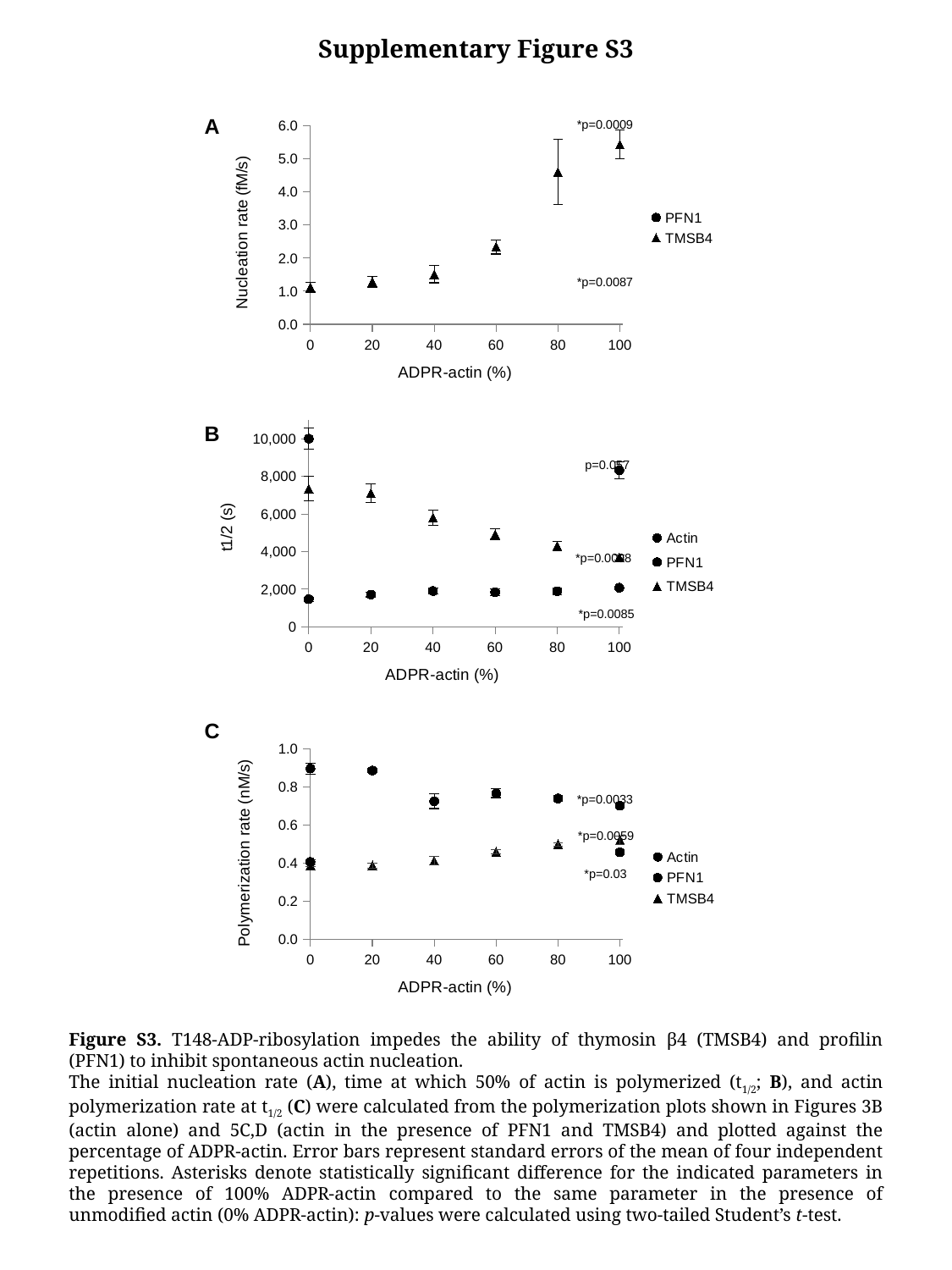

Supplementary Figure S3
A
*p=0.0009
### Chart
| Category | PFN1 | TMSB4 |
|---|---|---|*p=0.0087
B
### Chart
| Category | Actin | PFN1 | TMSB4 |
|---|---|---|---|p=0.057
*p=0.0098
*p=0.0085
C
### Chart
| Category | Actin | PFN1 | TMSB4 |
|---|---|---|---|*p=0.0033
*p=0.0059
*p=0.03
Figure S3. T148-ADP-ribosylation impedes the ability of thymosin β4 (TMSB4) and profilin (PFN1) to inhibit spontaneous actin nucleation.
The initial nucleation rate (A), time at which 50% of actin is polymerized (t1/2; B), and actin polymerization rate at t1/2 (C) were calculated from the polymerization plots shown in Figures 3B (actin alone) and 5C,D (actin in the presence of PFN1 and TMSB4) and plotted against the percentage of ADPR-actin. Error bars represent standard errors of the mean of four independent repetitions. Asterisks denote statistically significant difference for the indicated parameters in the presence of 100% ADPR-actin compared to the same parameter in the presence of unmodified actin (0% ADPR-actin): p-values were calculated using two-tailed Student’s t-test.

## Slide 5
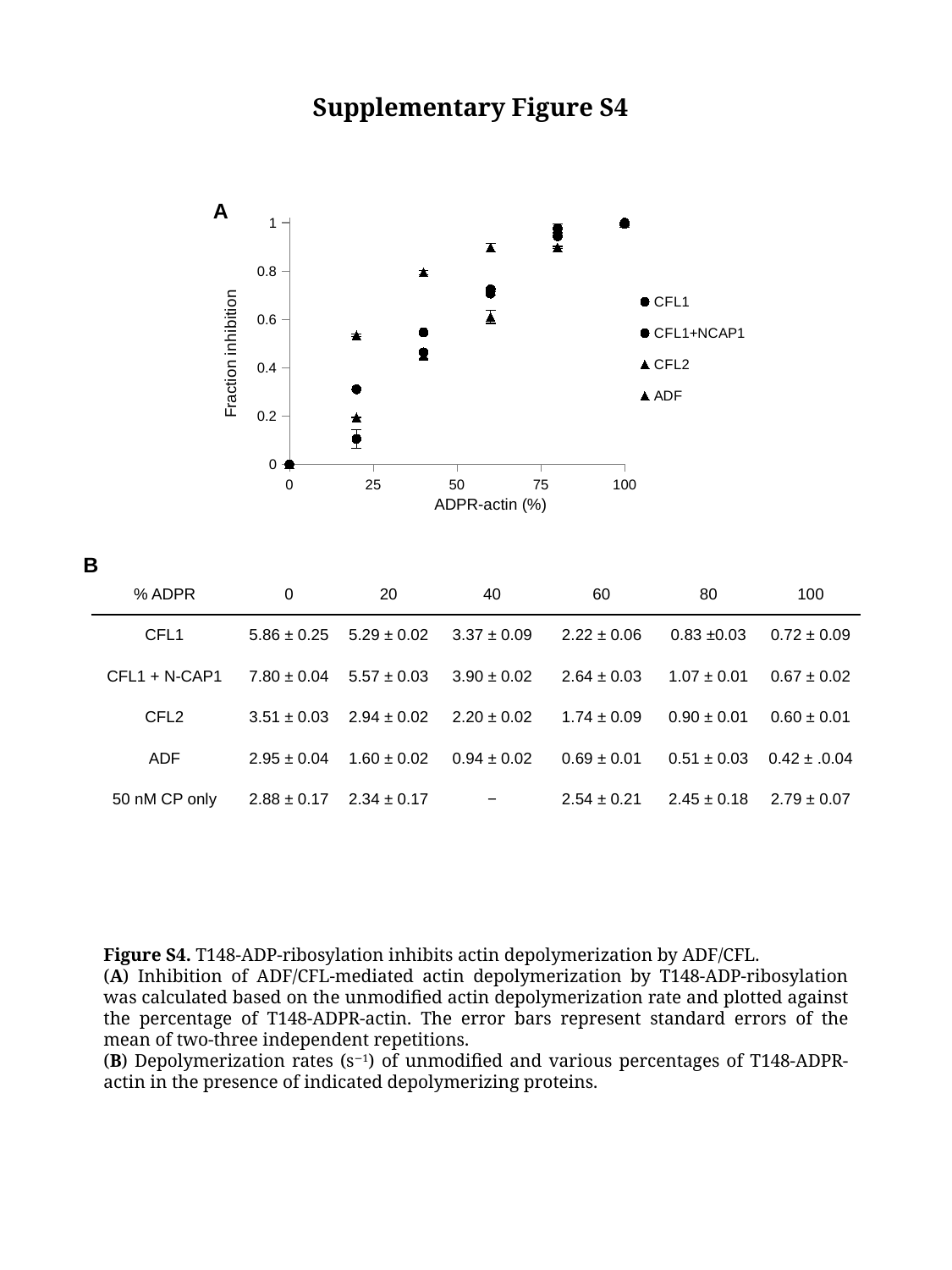

Supplementary Figure S4
A
### Chart
| Category | CFL1 | CFL1+NCAP1 | CFL2 | ADF |
|---|---|---|---|---|ADPR-actin (%)
B
| % ADPR | 0 | 20 | 40 | 60 | 80 | 100 |
| --- | --- | --- | --- | --- | --- | --- |
| CFL1 | 5.86 ± 0.25 | 5.29 ± 0.02 | 3.37 ± 0.09 | 2.22 ± 0.06 | 0.83 ±0.03 | 0.72 ± 0.09 |
| CFL1 + N-CAP1 | 7.80 ± 0.04 | 5.57 ± 0.03 | 3.90 ± 0.02 | 2.64 ± 0.03 | 1.07 ± 0.01 | 0.67 ± 0.02 |
| CFL2 | 3.51 ± 0.03 | 2.94 ± 0.02 | 2.20 ± 0.02 | 1.74 ± 0.09 | 0.90 ± 0.01 | 0.60 ± 0.01 |
| ADF | 2.95 ± 0.04 | 1.60 ± 0.02 | 0.94 ± 0.02 | 0.69 ± 0.01 | 0.51 ± 0.03 | 0.42 ± .0.04 |
| 50 nM CP only | 2.88 ± 0.17 | 2.34 ± 0.17 | − | 2.54 ± 0.21 | 2.45 ± 0.18 | 2.79 ± 0.07 |
Figure S4. T148-ADP-ribosylation inhibits actin depolymerization by ADF/CFL.
(A) Inhibition of ADF/CFL-mediated actin depolymerization by T148-ADP-ribosylation was calculated based on the unmodified actin depolymerization rate and plotted against the percentage of T148-ADPR-actin. The error bars represent standard errors of the mean of two-three independent repetitions.
(B) Depolymerization rates (s−1) of unmodified and various percentages of T148-ADPR-actin in the presence of indicated depolymerizing proteins.

## Slide 6
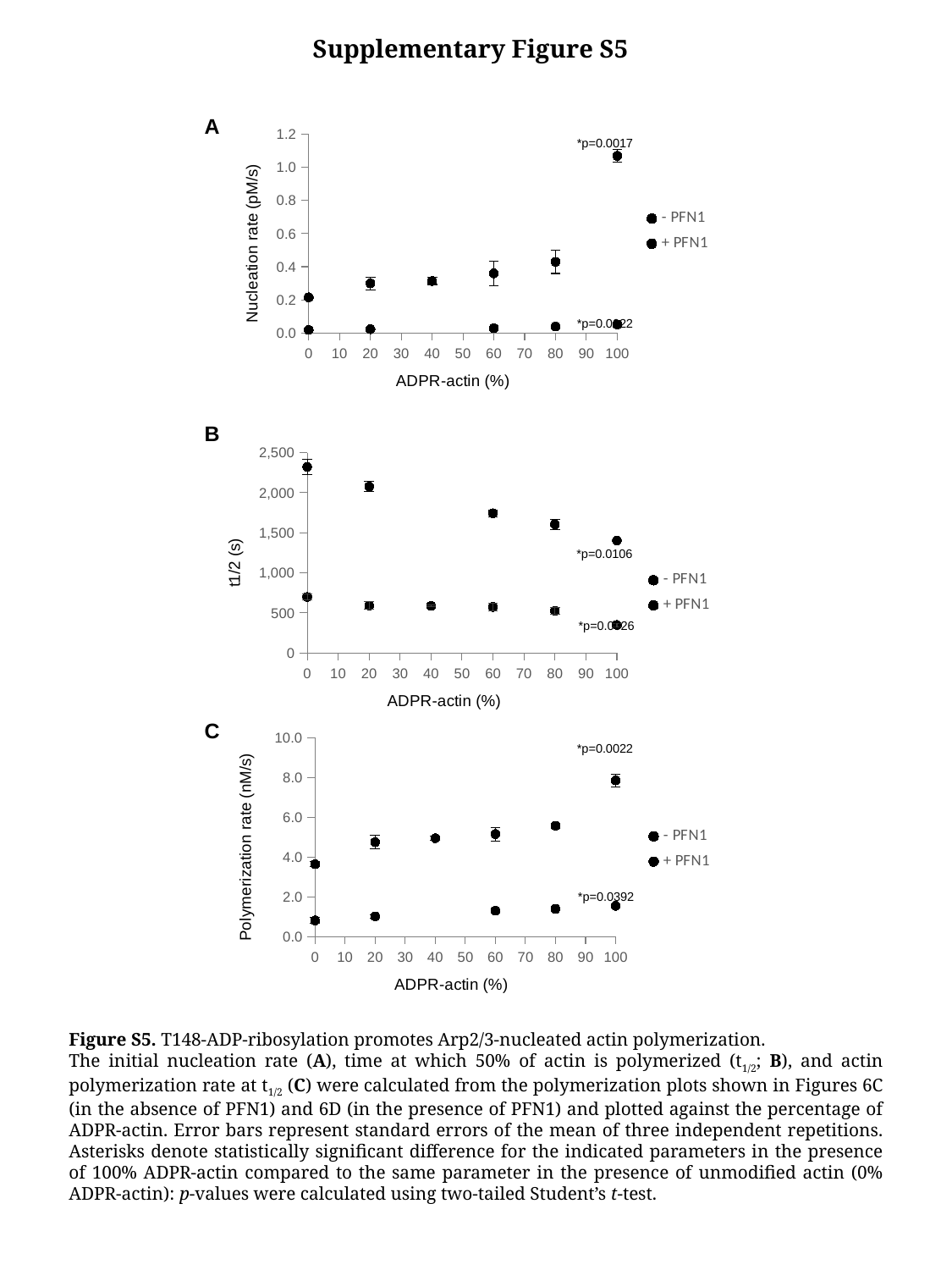

Supplementary Figure S5
A
### Chart
| Category | - PFN1 | + PFN1 |
|---|---|---|*p=0.0017
*p=0.0022
B
### Chart
| Category | - PFN1 | + PFN1 |
|---|---|---|*p=0.0106
*p=0.0026
C
### Chart
| Category | - PFN1 | + PFN1 |
|---|---|---|*p=0.0022
*p=0.0392
Figure S5. T148-ADP-ribosylation promotes Arp2/3-nucleated actin polymerization.
The initial nucleation rate (A), time at which 50% of actin is polymerized (t1/2; B), and actin polymerization rate at t1/2 (C) were calculated from the polymerization plots shown in Figures 6C (in the absence of PFN1) and 6D (in the presence of PFN1) and plotted against the percentage of ADPR-actin. Error bars represent standard errors of the mean of three independent repetitions. Asterisks denote statistically significant difference for the indicated parameters in the presence of 100% ADPR-actin compared to the same parameter in the presence of unmodified actin (0% ADPR-actin): p-values were calculated using two-tailed Student’s t-test.

## Slide 7
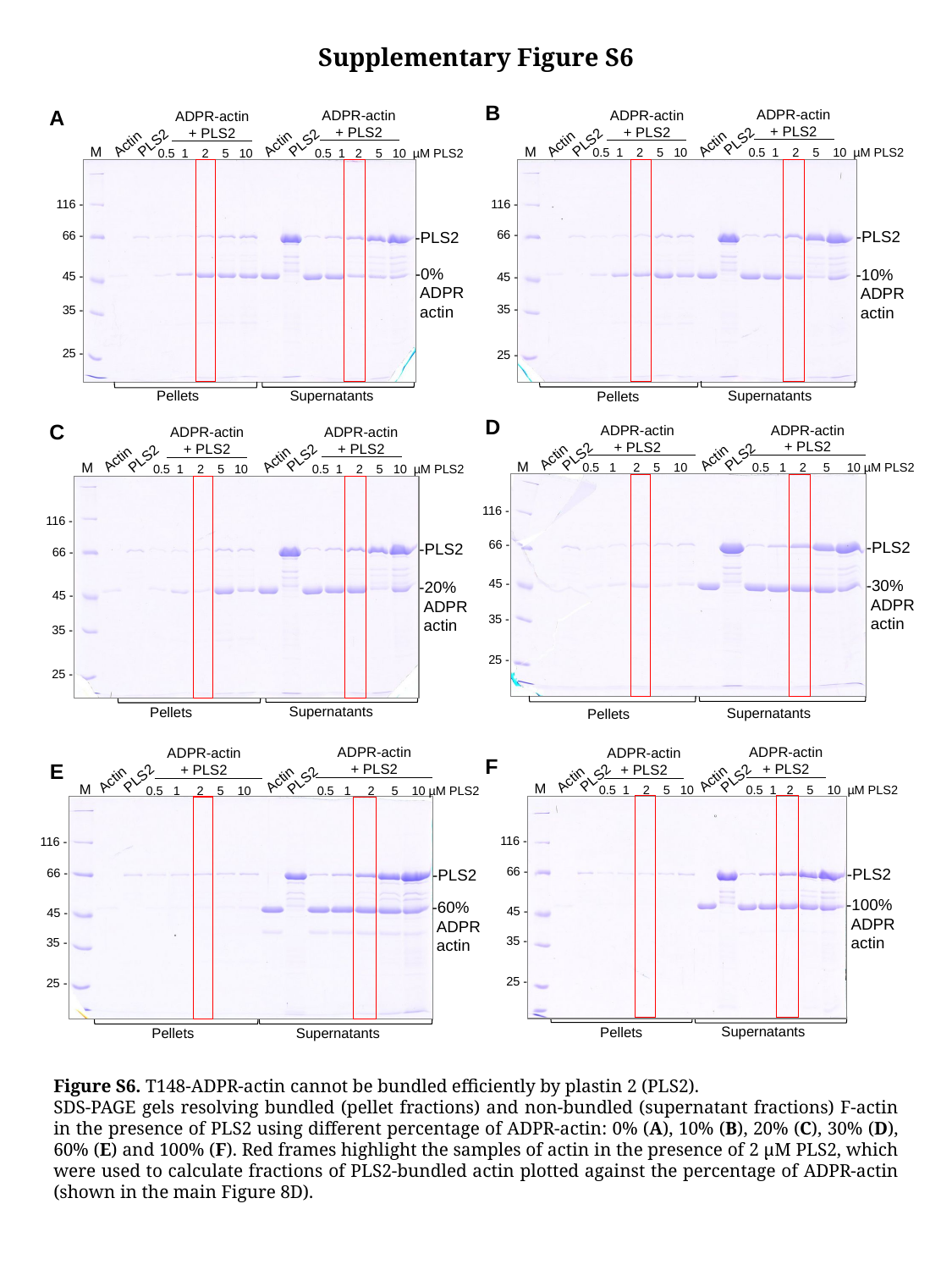

Supplementary Figure S6
B
A
ADPR-actin + PLS2
ADPR-actin + PLS2
PLS2
PLS2
Actin
Actin
M
0.5 1 2 5 10
0.5 1 2 5 10 µM PLS2
116 -
-PLS2
66 -
-10%
 ADPR
 actin
45 -
35 -
25 -
Supernatants
Pellets
ADPR-actin + PLS2
ADPR-actin + PLS2
PLS2
PLS2
Actin
Actin
M
0.5 1 2 5 10
0.5 1 2 5 10 µM PLS2
116 -
-PLS2
66 -
-0%
 ADPR
 actin
45 -
35 -
25 -
Supernatants
Pellets
D
C
ADPR-actin + PLS2
ADPR-actin + PLS2
PLS2
Actin
PLS2
Actin
M
 0.5 1 2 5 10
0.5 1 2 5 10 µM PLS2
116 -
66 -
-PLS2
-30%
 ADPR
 actin
45 -
35 -
25 -
Supernatants
Pellets
ADPR-actin + PLS2
ADPR-actin + PLS2
PLS2
Actin
PLS2
Actin
M
0.5 1 2 5 10
0.5 1 2 5 10 µM PLS2
116 -
-PLS2
66 -
-20%
 ADPR
 actin
45 -
35 -
25 -
Supernatants
Pellets
ADPR-actin + PLS2
ADPR-actin + PLS2
Actin
PLS2
PLS2
Actin
M
0.5 1 2 5 10
0.5 1 2 5 10 µM PLS2
116 -
66 -
-PLS2
-100%
 ADPR
 actin
45 -
35 -
25 -
Supernatants
Pellets
ADPR-actin + PLS2
ADPR-actin + PLS2
PLS2
Actin
Actin
PLS2
M
 0.5 1 2 5 10
0.5 1 2 5 10 µM PLS2
116 -
-PLS2
66 -
-60%
 ADPR
 actin
45 -
35 -
25 -
Supernatants
Pellets
F
E
Figure S6. T148-ADPR-actin cannot be bundled efficiently by plastin 2 (PLS2).
SDS-PAGE gels resolving bundled (pellet fractions) and non-bundled (supernatant fractions) F-actin in the presence of PLS2 using different percentage of ADPR-actin: 0% (A), 10% (B), 20% (C), 30% (D), 60% (E) and 100% (F). Red frames highlight the samples of actin in the presence of 2 µM PLS2, which were used to calculate fractions of PLS2-bundled actin plotted against the percentage of ADPR-actin (shown in the main Figure 8D).

## Slide 8
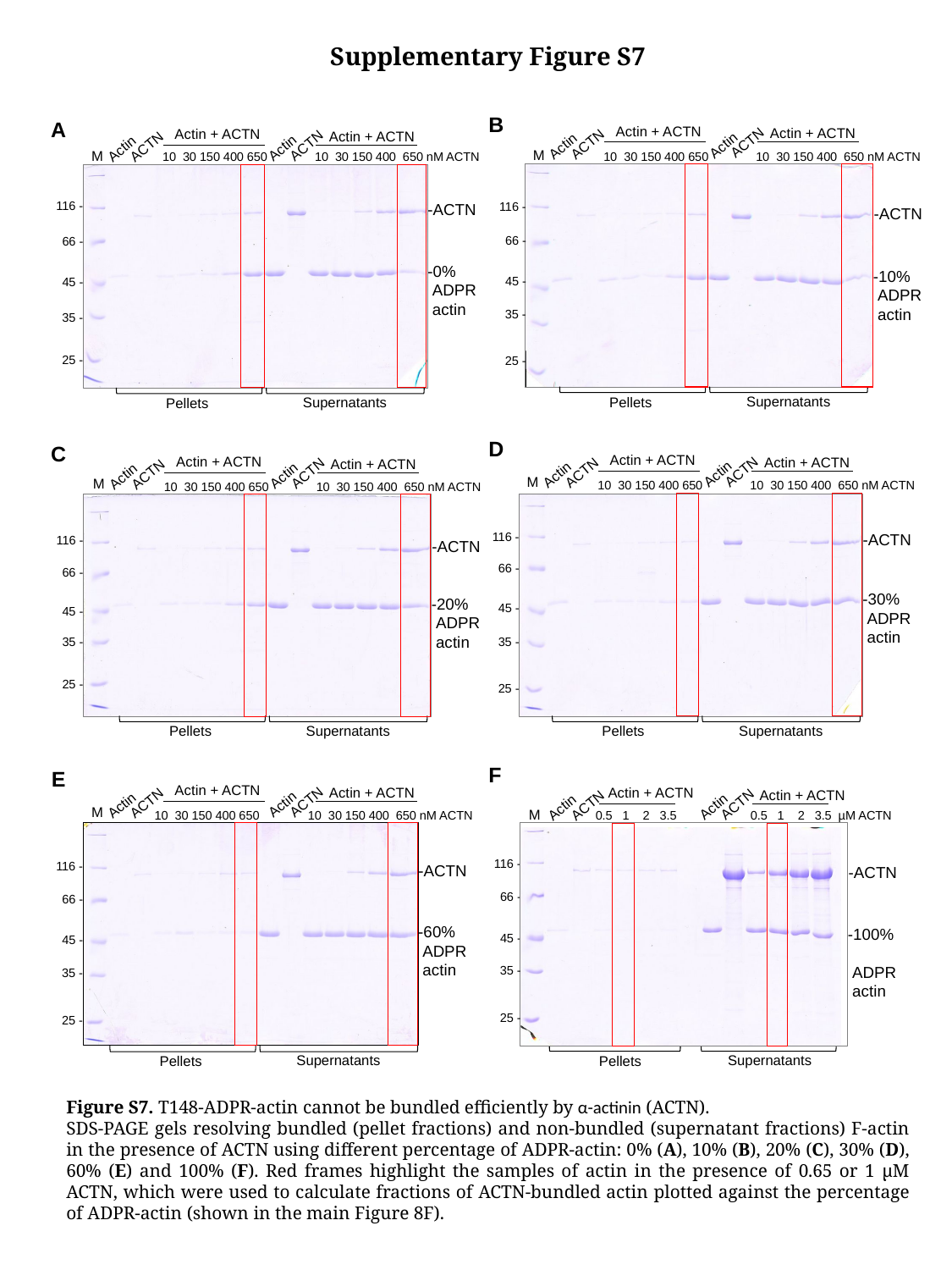

Supplementary Figure S7
B
A
Actin + ACTN
Actin + ACTN
Actin + ACTN
Actin + ACTN
ACTN
ACTN
Actin
Actin
ACTN
ACTN
Actin
Actin
M
M
10 30 150 400 650
10 30 150 400 650 nM ACTN
10 30 150 400 650
10 30 150 400 650 nM ACTN
116 -
116 -
-ACTN
-ACTN
66 -
66 -
-0%
 ADPR
 actin
-10%
 ADPR
 actin
45 -
45 -
35 -
35 -
25 -
25 -
Supernatants
Pellets
Supernatants
Pellets
D
C
Actin + ACTN
Actin + ACTN
Actin + ACTN
Actin + ACTN
ACTN
ACTN
Actin
Actin
ACTN
ACTN
Actin
Actin
M
M
10 30 150 400 650
10 30 150 400 650 nM ACTN
10 30 150 400 650
10 30 150 400 650 nM ACTN
116 -
-ACTN
116 -
-ACTN
66 -
66 -
-30%
 ADPR
 actin
-20%
 ADPR
 actin
45 -
45 -
35 -
35 -
25 -
25 -
Supernatants
Supernatants
Pellets
Pellets
F
E
Actin + ACTN
Actin + ACTN
Actin + ACTN
Actin + ACTN
ACTN
ACTN
Actin
Actin
ACTN
ACTN
Actin
Actin
M
M
0.5 1 2 3.5
0.5 1 2 3.5 µM ACTN
10 30 150 400 650
10 30 150 400 650 nM ACTN
116 -
116 -
-ACTN
-ACTN
66 -
66 -
-60%
 ADPR
 actin
-100%
 ADPR
 actin
45 -
45 -
35 -
35 -
25 -
25 -
Supernatants
Supernatants
Pellets
Pellets
Figure S7. T148-ADPR-actin cannot be bundled efficiently by α-actinin (ACTN).
SDS-PAGE gels resolving bundled (pellet fractions) and non-bundled (supernatant fractions) F-actin in the presence of ACTN using different percentage of ADPR-actin: 0% (A), 10% (B), 20% (C), 30% (D), 60% (E) and 100% (F). Red frames highlight the samples of actin in the presence of 0.65 or 1 µM ACTN, which were used to calculate fractions of ACTN-bundled actin plotted against the percentage of ADPR-actin (shown in the main Figure 8F).

## Slide 9
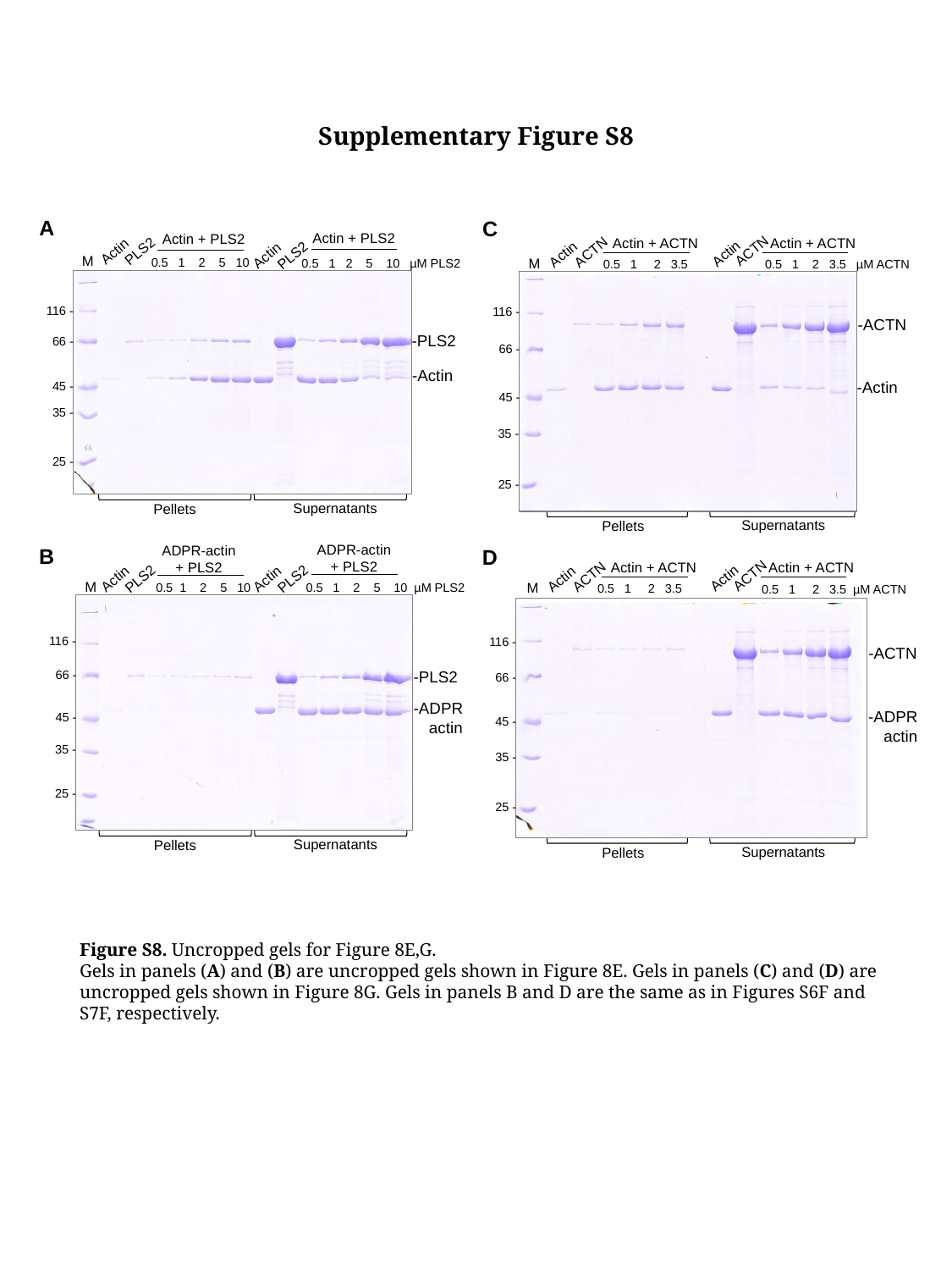

Supplementary Figure S8
A
C
Actin + PLS2
Actin + PLS2
Actin + ACTN
Actin + ACTN
PLS2
Actin
ACTN
ACTN
Actin
PLS2
Actin
Actin
M
M
0.5 1 2 5 10
0.5 1 2 5 10 µM PLS2
0.5 1 2 3.5
0.5 1 2 3.5 µM ACTN
116 -
116 -
-ACTN
-PLS2
66 -
66 -
-Actin
-Actin
45 -
45 -
35 -
35 -
25 -
25 -
Supernatants
Pellets
Supernatants
Pellets
ADPR-actin + PLS2
ADPR-actin + PLS2
B
D
Actin + ACTN
Actin + ACTN
ACTN
ACTN
PLS2
PLS2
Actin
Actin
Actin
Actin
M
M
0.5 1 2 5 10
0.5 1 2 5 10 µM PLS2
0.5 1 2 3.5
0.5 1 2 3.5 µM ACTN
116 -
116 -
-ACTN
-PLS2
66 -
66 -
-ADPR
actin
-ADPR
actin
45 -
45 -
35 -
35 -
25 -
25 -
Supernatants
Pellets
Supernatants
Pellets
Figure S8. Uncropped gels for Figure 8E,G.
Gels in panels (A) and (B) are uncropped gels shown in Figure 8E. Gels in panels (C) and (D) are uncropped gels shown in Figure 8G. Gels in panels B and D are the same as in Figures S6F and S7F, respectively.
